# Testing the Optimal Foraging Theory in a Generalist Feeder: The Case of Reintroduced European Pond Turtles and Its Impact on Macroinvertebrates Communities

**DOI:** 10.1002/ece3.71823

**Published:** 2025-08-13

**Authors:** Albin Meyer, Corinne Grac, Frédéric Labat, Johannes Meka, Karina A. E. van der Zon, Kathrin Theissinger, Jean‐Yves Georges

**Affiliations:** ^1^ INRAE, UR EABX Cestas France; ^2^ Université de Strasbourg, CNRS, ENGEES, LIVE UMR 7362 Strasbourg France; ^3^ AQUABIO Clermont‐Ferrand France; ^4^ Université de Strasbourg, CNRS, IPHC UMR 7178 Strasbourg France; ^5^ Rheinland‐Pfälzische Technische Universität (RPTU) Kaiserslautern‐Landau, Faculty of Biology, Ecology Department Kaiserslautern Germany; ^6^ Long‐Term Studies in Ecology and Evolution (SEE‐Life) program of CNRS, Neu Woerr, Lauterbourg & Neuburg Am Rhein Strasbourg France

**Keywords:** biological trait, eDNA metabarcoding, *Emys orbicularis*, reintroduction, trophic interaction

## Abstract

In the context of biodiversity erosion, species reintroductions are considered a promising strategy for limiting species extinction. However, the impacts of introducing a formerly extinct species in current ecosystems are poorly reported in terms of ecosystem functioning and trophic ecology, especially in the case of reintroduced generalist feeders. Based on the optimal foraging theory, we tested the prediction that the generalist European pond turtle ( *Emys orbicularis* ) feeds on a wide range of prey, yet optimises its energy intake by targeting larger and/or softer prey. We characterised the diet of captive‐bred turtles once released on the Woerr site, Upper Rhine Valley, North East of France. eDNA metabarcoding was implemented on faecal samples from 15 subadult individuals, with a focus on consumed macroinvertebrates (MI) that are reported to be the major prey of the species. Furthermore, we investigated the temporal trends of the MI community throughout successive turtle releases over 5 years, in order to assess the consequences of the turtle reintroduction on ecosystem functioning. Faecal eDNA analyses revealed that after their release turtles exhibited a highly diversified diet (insects, gastropods, but also plants and amphibians). Importantly, turtles showed a preference for prey with relatively large potential body size and high longevity (Odonata, Coleoptera and Hemiptera). Yet, the successive releases of turtles did not impact the MI community over time. We conclude that reintroduced European pond turtles operate as a new top predator in the system, with larger turtles potentially feeding on larger prey, as predicted by their opportunistic generalist feeding ecology, yet without changing the overall community of MI, most likely due to the low predation pressure they exert on the prey community. This study highlights the relevance of conservation initiatives such as species reintroductions benefiting local biodiversity without jeopardising existing ecosystem functioning.

## Graphical abstract

In this study we investigated the macroinvertebrate (MI) communities in two newly created, man‐made ponds concurrently with the diet of captive‐bred European pond turtles that were translocated there. We aimed at assessing whether turtles were operating as generalist feeders, and if successive reintroductions of turtles would affect the overall MI communities, by testing the prediction based on the optimal foraging theory that turtles would select larger prey taxa for optimizing their food intake. We conclude that reintroduced European pond turtles operate as a new top predator in the system, with larger turtles potentially feeding on larger prey, as predicted by their opportunistic generalist feeding ecology, yet without changing the overall community of MI, most likely due to the low predation pressure they exert on the prey community.

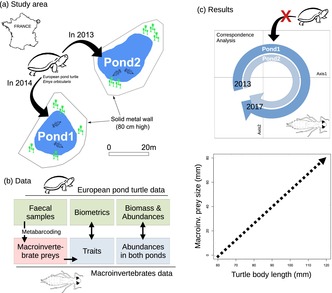

## 1 Introduction

In the present context of the 6th crisis of species mass extinction mostly due to human‐induced destruction, degradation, fragmentation and pollution of natural habitats (Cowie et al. 2022), habitat restoration and reintroduction of threatened species are considered to be an operational strategy for limiting biodiversity erosion (Sarrazin and Barbault 1996). Species reintroductions consist of releasing (usually captive‐bred) individuals of a species in their formerly natural habitat (IUCN 2013). As such, the success of these conservation actions strongly depends on the intrinsic capabilities of individuals to survive and reproduce in the wild, especially when they were raised in captive, usually highly artificial, conditions (Quintard and Georges 2023). Most importantly, reintroductions have been reported to alter community structure, composition and ecosystem functions (Alston et al. 2019; Byrne and Pitchford 2016; Genes et al. 2019). For instance, reintroducing predators has been shown to trigger trophic cascades via changes in prey and competitor densities and behaviors (Bakker and Svenning 2018; Cunningham et al. 2019; Ripple et al. 2014; Scoleri et al. 2023; Svenning et al. 2016). Nevertheless, most species reintroductions tend to focus on large species (e.g., carnivore mammals and birds; Gilbert et al. 2017), but only to a lesser extent on smaller carnivores or omnivorous species. As a consequence, the impacts of introducing omnivorous species of lower trophic levels are poorly reported, especially in terms of ecosystem functioning.

The European pond turtle *Emys orbicularis* (Linnaeus, 1758) is a small‐sized freshwater turtle distributed throughout Europe from the Iberic peninsula to Baltic countries and Ukraine, and to a lesser extent in Northern Africa (Fritz and Chiari 2013; Nekrasova and Marushchak 2023). The species has suffered the most dramatic decline of all reptiles in Europe mainly due to wetland mismanagement (Fritz and Chiari 2013; Liuzzo et al. 2024). The species has even become extinct in several regions due to anthropogenic pressures, including former human consumption (Schneeweiss 1997; as reported by Raemy and Ursenbacher 2018), and more recently habitat loss (Ficetola et al. 2004), pollution (Guillot et al. 2018), as well as trophic competition with invasive exotic species, such as the red‐eared slider *Trachemys scripta elegans* (Wied, 1839) (Balzani et al. 2016). To counter its decline, the European pond turtle has benefited from numerous conservation initiatives throughout Europe over the last decades (reviews in Fritz and Chiari 2013; Cordero‐Rivera et al. 2024; Georges et al., in prep–a). During soft reintroductions, captive‐bred individuals are first released in open sky fenced ponds, where they can acclimate to near‐natural conditions for several months while being protected from predation before enabling them to disperse freely (Quintard and Georges 2023; Ottonello et al. 2010). As such, acclimation is a crucial phase both for the released population and also for scientists, since it facilitates the monitoring of the released individuals (Quintard and Georges 2023; Georges et al., in prep–b). Previous monitoring studies have reported that reintroduced turtles can exhibit satisfying survival rates, while showing diet and behaviour similar to those observed in wild populations (Canessa et al. 2016; Cordero‐Rivera et al. 2024; Escoriza et al. 2020; Ottonello et al. 2018).

The European pond turtle is an opportunistic omnivorous species (Ducotterd et al. 2020). Based on the analyses of prey remains or eDNA in faecal samples, the species has been shown to feed mainly on macroinvertebrate (MI) prey, mainly Odonata, Hemiptera, Coleoptera, Ephemeroptera, and Diptera, but also Plecoptera, Hymenoptera, Trichoptera, Oligochaeta, Amphipoda, Isopoda, Hexapoda, and Gastropoda (Ducotterd et al. 2020; Ottonello et al. 2018). Yet, it also can feed on plants (Ayres et al. 2010; Ducotterd et al. 2020; Ottonello et al. 2018) and vertebrates (including amphibians and dead fish, Çiçek and Ayaz 2011; Vergilov et al. 2021). As shown for Emydidae, the diet can vary through ontogeny, with juveniles exhibiting a carnivorous diet whereas adults preferentially feed on plants (Ottonello et al. 2005). Adults may also seasonally shift their diet from invertebrates in spring–summer to mostly plants and vertebrates in July–September, as reported in Turkey (Çiçek and Ayaz 2011). Yet, contrasting results have been reported from Switzerland, where no differences in the diet of captive turtles have been shown between neither sexes nor age classes, despite adults exhibiting a more diverse diet during the reproduction period (Ducotterd et al. 2020). Overall, these previous studies suggest a highly opportunistic feeding behaviour of the European pond turtle. However, its functional roles within the freshwater food web in the context of the optimal foraging theory (MacArthur and Pianka 1966) have been poorly investigated.

According to the optimal foraging theory (MacArthur and Pianka 1966), generalist species are predicted to feed on a wide range of prey that either permits them to optimise their energy intake (by targeting larger prey) and/or reduce the prey handling time (by targeting soft prey). Besides, larger prey may only be accessible to larger turtles, due to prey–predator relative swimming abilities and/or due to the incapacities of smaller turtles to feed on hard pieces of larger prey. In this study, we aimed at monitoring the trophic ecology of European pond turtles of different body sizes at their release in the Rhine floodplain and the consequences of the turtle reintroduction on present MI communities. The reintroduction consisted of successive soft releases of tens of juvenile to subadult turtles in two acclimatisation ponds (Quintard and Georges 2023; Georges, Priol, et al., in prep–b). With genetic analyses of the turtles' feces, we aimed at testing the optimal foraging theory by predicting that (1) larger turtles should be able to feed on larger chitinised prey items (e.g., insects and gastropods) while (2) larger densities of turtles resulting from successive releases should affect the MI community (taxonomic composition and abundance) by benefiting species with smaller body sizes and faster paces of life. The underlying goal of this study was to assess the ability of the newly created acclimatisation ponds to sustain the reintroduced population of European pond turtles.

## 2 Materials and Methods

### 2.1 Study Area

**FIGURE 1:**
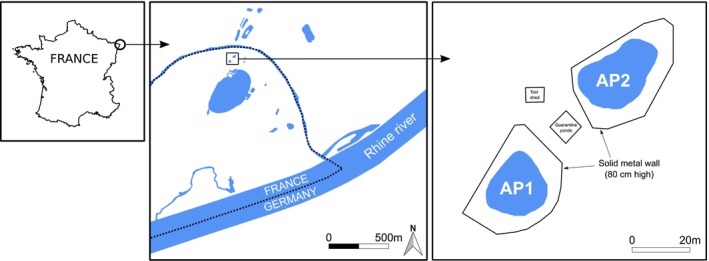
Location of the Woerr site and of the two acclimatisation ponds (AP).

The study was conducted on the Woerr site, Lauterbourg, NE of France (lon. 8.2208°, lat. 48.9751°), where the European pond turtle benefits from reintroduction initiatives since 2000 led by the local council Collectivité européenne d'Alsace (CeA) (Philippot and Georges 2023) (Figure 1). Formerly used for gravel extraction until 1994, the Woerr site benefited from successive restoration actions and nowadays consists of a 72 ha protected area co‐managed by the French National Office of the Forests and by the CeA (see Philippot and Georges 2023). As part of restoration and reintroduction actions, an acclimatisation site was created in 2013. The 1000 m^2^ site, surrounded by wildlife fences for preventing predation of turtles, encompasses two 300 m^2^ artificial ponds (hereafter referred as acclimatisation ponds AP1 and AP2, 150 cm depth) watered with ground water thanks to a system of pumps (see Quintard and Georges 2023). Each AP is surrounded by an 80 cm high solid metal wall preventing turtles from exiting except from 5 openings that can be manually manipulated to permit turtles to disperse freely. Both APs were artificially planted with local aquatic species including reeds.

### 2.2 Monitoring of the MI Communities

From 2013 to 2017, the MI communities of both acclimation ponds were sampled once a year, in June–July. The sampling method was the prototype of protocol S3m (Labat et al. 2022), developed for the sampling of MIs in small shallow lakes and ponds. Mesohabitats were defined according to their suitability for MIs. For each vegetated mesohabitat, a 1 m^2^ plot was sweep‐net sampled, whereas in each non‐vegetated mesohabitat, a 1/20 m^2^ plot was kick‐net sampled. Samples were evenly located in all available mesohabitats. For each pond, 12 incremental samples were taken, i.e., when there were less than 12 mesohabitats, replicates were taken proportionally and respectively to the area occupied by each mesohabitat. Samples were preserved in alcohol (~70%) until taxonomic determination in the laboratory. There, MI individuals were manually sorted and identified to the lowest taxonomic level possible, usually to the genus level, and to the family level for Diptera and Odonata, following Labat (2017).

In order to describe the structure of the MI community, four standard metrics were calculated for each sampling event: the taxonomic richness (S), total abundance (ind./m²), Shannon's index (*H*′; Shannon, 1948) and Pielou's evenness (*E*). Shannon's index is an index measuring taxonomic diversity, and it was calculated using the following formula:

$$H^{\prime }=-\sum_{i}p_{i}\log_{2}p_{i}$$

where $p_{i}$ is the relative abundance of taxon *i* for a given sampling event. Pielou's evenness (*E*) is a measure of the evenness of the MI community and it is derived from *H*′, by dividing it by its theoretical maximal value:

$$E=H^{\prime }/\log_{2}S$$

where *S* is the taxonomic richness of a given sampling event.

### 2.3 Analysis of MI Traits

For the MI dataset of 2015, when turtles' faeces were sampled, an analysis of 11 biological traits of the 76 MI taxa found in the acclimatisation ponds and/or in the faeces was performed. The prey traits were selected based on available data (from Tachet et al. 2010) and were: ‘Maximal size’, ‘Life cycle duration’, ‘Potential number of reproductive cycles per year’, ‘Aquatic stages’, ‘Reproduction’, ‘Dispersal’, ‘Resistance form’, ‘Respiration’, ‘Locomotion and substrate relation’, ‘Food’, ‘Feeding habits’. The 11 prey traits were described by 63 categories, either a qualitative nominal or quantitative ordinal category, and analysed following the fuzzy coding procedure described in Usseglio‐Polatera et al. (2000) (Table S1). Qualitative traits were described with one modality for each nominal category (e.g., ‘aerial, passive’, ‘aerial, active’, ‘aquatic, passive’, and ‘aquatic, active’ for the trait ‘Dispersal’). Quantitative traits were described with one modality for each ordinal category. For example, for the trait ‘Maximal potential size’, there is one modality for each size class, i.e., ‘< 0.25 cm’, ‘≥ 0.25–0.5 cm’, ‘≥ 0.5–1 cm’, ‘≥ 1–2 cm’, ‘≥ 2–4 cm’, and ‘≥ 4–8 cm’. The affinity of each taxon to each modality was assigned using scores ranging from 0 (*modality not observed for a given taxon*) to 5 (*modality always observed*) (Usseglio‐Polatera et al. 2000). For MI taxa identified to family levels, the affinity scores were the averages of the scores for the genera belonging to this given family and described in Tachet et al. (2010).

The affinity scores for the family Aeshnidae (Anisoptera) were refined by only using the affinity scores of genera belonging to this family and observed either as larvae in the pond samples or as adults on the site in 2015 (namely: *Aeshna*, *Anax* and *Brachytron*; ECOLOR 2015). In a similar fashion, although Coenagrionidae individuals were only identified at the family level during the monitoring of the MI communities (see previous section), the traits information of both Coenagrionidae taxa identified in the faecal samples, i.e., the genera *Coenagrion* and *Ischnura*, were actually used for further trait analyses. Such a refinement was only applied to these two families due to their potential importance, as observed in the turtles' faeces.

Before further calculations, the affinity scores for each pair [trait × taxon] were expressed as relative affinities, i.e., affinity scores were divided by the sum of the scores of all modalities belonging to a given trait. For example, for a trait with three modalities and a taxon with affinity scores equal to 0, 2 and 3 for each modality, respectively, the new trait profile would be 0, 0.4 and 0.6. Eight rare trait modalities, exhibiting a low average relative affinity among all taxa (less than 1.2%), were removed before further analyses.

### 2.4 Turtles and Faecal Samples Collection

From 2013 to 2017, 82 European pond turtles born and raised in captivity were released at ages ranging from 3 to 7 years in the Woerr APs during three successive release events (Table 1). Before the last release event, turtles that survived in AP2 were translocated to AP1. All individuals were tagged using implanted transponders (OIN FDX‐B, Planet ID, https://www.planet‐id.com/) and with small notches made on marginal scales of the carapace using a small metal file (see Quintard and Georges 2023).

**TABLE 1:** Reintroduction events and basic biometry data at translocation time of the European pond turtles on Woerr site, France, between 2013 and 2017.

Date of reintroduction	Translocation (from ➔ to)	No. of translocated individuals	Age (years, $\bar{x}$ ± SE [min–max])	Body length (mm, $\bar{x}$ ± SE [min–max])	Body mass (g, $\bar{x}$ ± SE [min–max])
10 Oct 2013	Captivity➔AP2	15	6 ± 1 [6–7]	96 ± 11 [75–114]	164 ± 58 [79–263]
6 Aug 2014	Captivity➔AP1	23	5 ± 1 [4–6]	69 ± 6 [57–80]	66 ± 19 [38–108]
26 June 2016	AP2➔AP1	13	10 ± 1 [9–10]	—	275 ± 55 [190–340]
28 June 2016	Captivity➔AP2	43	4 ± 0 [4–4]	55 ± 7 [40–79]	37 ± 15 [18–90]

In June 2015, faecal samples were collected from the 15 subadult individuals that were released at 6–7 years old at the first event. Every captured individual was identified, measured using callipers (carapace length and width; ±0.1 mm) and weighed using a spring scale (body mass; ±0.1 g). Once measured, they were placed in an individual plastic box (40 × 30 × 30 cm) in which a metal mesh was placed 1 cm above the bottom. This permitted the collection of faeces without risking faecal pellet crushing by the turtle. Each individual was checked twice a day to ensure its well‐being and in order to collect fresh faeces. After the faeces collection, the turtle was released near the capture location. Each faecal sample was placed in two imbricated plastic bags and placed in a freezer (−20°C) until analyses.

### 2.5 eDNA Metabarcoding

A metabarcoding approach was used to detect and identify traces of food items in the faecal samples (e.g., Taberlet et al. 2012). The analyses were performed by SPYGEN (Le Bourget‐du‐Lac, France). The DNA extraction was performed following the protocol described in Valentini et al. (2009). After extraction from the faecal samples, DNA was amplified using three pairs of universal primers: one for plants (Sper01; Taberlet et al. 2007; Taberlet et al. 2018), one for insects (Inse01; Elbrecht et al. 2016; Taberlet et al. 2018), and one for amphibians (Batr01; Taberlet et al. 2018; Valentini et al. 2016). The amplification was carried out in a final volume of 25 μL, using 3 μL of DNA extract as a template, following the protocol described in Valentini et al. (2016), but human blocking primer was only used for amphibian DNA amplification. The PCR mixture was denatured at 95°C for 10 min, followed by 40 cycles of 30 s at 95°C, 30 s at 55°C for Batra01, 50°C for Sper01, and 56°C for Insec01 and 1 min at 72°C, followed by a final elongation at 72°C for 7 min in a room dedicated to amplified DNA with negative air pressure and physical separation from the DNA extraction rooms (with positive air pressure). Two PCR replicates were performed per sample. Two replicates of the negative extraction control and PCR control (ddH_2_O) were amplified and sequenced in parallel. After amplification, the samples were purified using a MinElute PCR purification kit (Qiagen, GmbH) and quantified using capillary electrophoresis (QIAxcel; Qiagen GmbH). The purified PCR products were pooled in equal volumes to achieve an expected sequencing depth of 50,000 reads per sample. Library preparation and sequencing were performed at the Fasteris facility (Geneva, Switzerland). The library was prepared using the Metafast protocol (https://www.fasteris.com/). The library was sequenced using Illumina HiSeq (2 × 125 bp; Illumina, San Diego, CA, USA), following the manufacturer's instructions.

### 2.6 Bioinformatic Processing

The sequence reads were analysed using programmes implemented in the OBITools package (Boyer et al. 2016), following a protocol described in Valentini et al. (2016). The forward and reverse reads were assembled using the illuminapairedend program, by using a minimum score of 40 and by retrieving only joined sequences. The reads were then assigned to each sample using the ngsfilter program. Strictly identical sequences were clustered together by using obiuniq. Sequences shorter than 10 bp, or with occurrences lower than 10 reads, or labelled ‘internal’ by the obiclean program were excluded. Taxonomic assignment was performed using the ecotag program, with the sequences extracted from ENA Release 123 (standard sequences) of the European Bioinformatics Institute's EMBL database using the ecoPCR program (Ficetola et al. 2010) or a local sequence database for amphibians (Valentini et al. 2016). Only sequences showing a similarity higher than 98% with the reference database were retrieved.

### 2.7 Statistical Analyses

**FIGURE 2:**
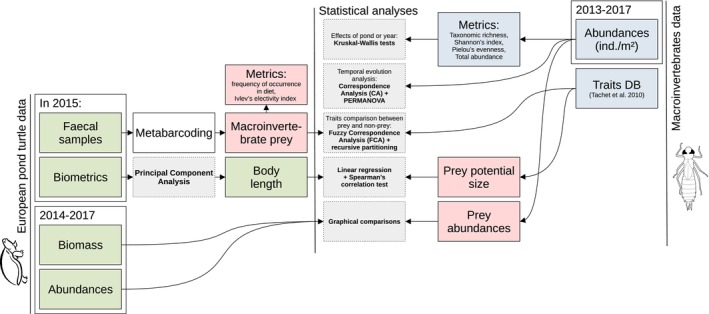
Overview of the studied data and statistical analyses.

Statistical analyses were carried out with the program R (v4.2.1; R Core Team 2022). The following packages were used: vegan (Oksanen et al. 2020) and ade4 (Dray and Dufour 2007) for metric calculations and multivariate analyses; selectapref (Richardson 2018) for calculating Ivlev's electivity index; partykit (Hothorn and Zeileis 2015) for the recursive partitioning. Values are given as mean ± SD and the significance threshold was set to 5%. Figure 2 summarizes acquired data and statistical analyses.

#### 2.7.1 Macroinvertebrate Community in the Ponds

The dataset containing the abundance of MI taxa in each pond for each year [85 taxa × 2 ponds × 5 years] was analyzed with a Correspondence Analysis (CA; Benzécri 1976) in order to assess how the structure (composition and abundance) of the MI community changed over time in relation to the reintroduction events in both ponds (Table 1). Abundance data were log(*x* + 1)‐transformed prior to the analysis, and rare taxa (i.e., taxa only observed once, all samples combined) were excluded from the dataset. The CA was followed by a variance partitioning to identify the importance of the effects of the pond and of the year on the observed ordination. The variance partitioning was completed by permutational multivariate analyses of variance (PERMANOVA), allowing us to test whether the pond or the sampling year had a significant effect on the community structure. The PERMANOVA tests were performed separately for ponds and years. They were performed on the log(*x* + 1)‐transformed abundance dataset, using the Bray–Curtis distance to obtain the pairwise dissimilarity matrix between each sampling event (pond × year). The effects of pond and year on the values of the structure of the MI community (*S*, *H*′ and *E*) were tested with Kruskal–Wallis tests, as conditions for ANOVA tests (normality and homoscedasticity) were not met.

The array containing the traits data [76 taxa × 55 trait categories] was analyzed with a fuzzy correspondence analysis (FCA; Chevenet et al. 1994). In order to assess whether or not prey exhibited specific biological traits, a recursive partitioning was used to assess if the positions of potential prey and non‐prey significantly differed along the factorial axes of the FCA (Strobl et al. 2007).

#### 2.7.2 Prey Data

Prey were coded as present or absent from faecal samples. In order to evaluate potential prey selection by the pond turtles, two metrics were calculated based on this data: the Frequency of Occurrence (FO) was expressed as the number of faeces containing the potential prey divided by the total number of faeces (*n* = 15) and Ivlev's electivity index (IEE) (Ivlev 1961) was calculated for each prey (i) as IEEi = (ri—pi)/(ri + pi), where ri is the percentage of faeces containing the prey (i) and pi its frequency in the environment (i.e., the average proportion of the prey (i) in the MI communities of AP1 and AP2). Values of IEE range from −1 to 1, which correspond respectively to avoided or preferred prey items (IEE = 0 in case of random feeding).

#### 2.7.3 Turtle Biometric Data

The dataset containing the biometrics of the captured pond turtles which faecal samples was analysed by a normalised Principal Component Analysis (nPCA; Figure S1). Since turtle biometrics data were highly correlated, we simply used carapace length as a body size index for assessing the relation between turtle body size and the size of prey found in turtles´ faeces.

## 3 Results

### 3.1 Bioinformatic Processing of Raw Data

In total, 1,393,113 reads were obtained for plants (99,508 ± 89,161 per sample), 11,533,582 reads for insects (768,905 ± 1,006,486 per sample) and 2,774,704 reads for amphibians (184,980 ± 133,886 per sample). After the bioinformatics steps, 1,135,970 reads (81.5%) were retrieved for plants (81,141 ± 76,674 per sample), 2,516,747 reads (21.8%) for insects (167,783 ± 438,582 per sample) and 476,865 reads (17.2%) for amphibians (31,791 ± 47,734 per sample). In total, 65 plant taxa, 11 insect taxa, and one amphibian taxon were identified in the faecal samples.

### 3.2 MIs Communities Before and After Turtle Releases

In 2013, i.e., before the first release event, the composition of the MI communities found in both APs was quite similar (Table 2). Community compositions were equally represented by taxa belonging to Coleoptera (taxonomic richness *S* = 12 for both APs), Diptera (*S* = 8 and 10, respectively for AP1 and AP2), Heteroptera (*S* = 6 and 7, respectively), Odonata (*S* = 7 and 6, respectively) and Gastropoda (*S* = 8, for both APs). Diptera densities were higher in AP2 than in AP1, with Diptera representing 57.0% and 32.4% of the total density, respectively in AP2 and AP1. Diptera were mainly represented by taxa belonging to four families: Ceratopogonidae, Chaoboridae, Chironomidae, and Stratiomyidae. Gastropods were also quite abundant in both APs, with the most abundant genera being *Physella* and *Gyraulus*. Other abundant taxa were insects, such as Coleoptera (*Helochares* and *Hydroglyphus* in both APs, *Enochrus* in AP2), Heteroptera (*Gerris* in both APs, *Microvelia* in AP2) and Odonata (Coenagrionidae, *Orthetrum*, *Sympecma* and *Sympetrum* in both APs, Aeshnidae in AP2). Bivalvia belonging to the family Sphaeriidae were also quite abundant in AP2. Both AP1 and AP2 showed similar taxonomic richness (49 and 50 taxa, respectively), but with varying diversities, with Shannon's index equal to 2.56 and 3.19, respectively, and Pielou's evenness equal to 0.46 and 0.56, respectively. However, MI were ~3 times less abundant in AP1 than in AP2 (total abundances equal to 158,314 versus 517,646 ind./m², respectively) while surface areas were similar between the two ponds. Total abundance differences between ponds were mainly due to Diptera, Ephemeroptera, Heteroptera, and Odonata that were about 5–13 times more abundant in AP2 than in AP1 (Table 2).

**TABLE 2:** Main characteristics of the macroinvertebrate assemblages found each year in each pond, and densities of the 40 most abundant taxa (ind./m^2^).

	Pond	AP1	AP1	AP1	AP1	AP1	AP2	AP2	AP2	AP2	AP2
	Sampling year	2013	2014	2015	2016	2017	2013	2014	2015	2016	2017
	Taxonomic richness	49	54	55	53	45	50	54	63	46	45
	Total abundance (ind./m²)	158,314	42,527	50,403	16,212	22,170	517,646	32,372	31,872	8436	26,175
	Shannon's index	2.56	2.85	3.16	3.22	2.81	3.19	3.15	3.77	4.00	2.99
	Pielou's evenness	0.46	0.49	0.55	0.56	0.51	0.56	0.55	0.63	0.72	0.54
Taxonomic group	Taxa										
Coleoptera	*Enochrus* ^a^	0	0	55	0	120	5953	0	99	0	0
	*Helochares*	3799	231	517	170	8	380	285	74	58	0
	*Hydrochara*	3	88	520	4	16	13	5	0	0	36
	*Hydroglyphus*	1453	143	0	0	0	1038	68	0	0	0
	*Hyphydrus* ^a^	12	376	10	306	0	0	32	0	236	63
	*Laccobius*	895	0	0	28	0	13	0	0	0	12
	*Laccophilus*	40	1748	762	24	404	174	1329	1499	1	387
	*Rhantus*	63	95	422	0	12	133	5	381	0	24
Diptera	Ceratopogonidae	1530	11	15	22	4	54,546	73	91	23	0
	Chaoboridae	4831	3430	1435	3750	4498	35,841	4269	6057	1760	3890
	Chironomidae	25,066	8999	3292	1484	190	190,166	8142	3937	701	292
	Culicidae	53	14	1091	36	1026	1522	58	5071	109	369
	Limoniidae	7	0	0	0	0	1571	48	46	0	0
	Stratiomyidae	19,548	413	1328	734	196	11,149	1138	3087	481	108
	Tabanidae	0	0	433	0	4	0	0	257	0	0
Ephemeroptera	*Caenis*	7270	211	238	34	88	46,454	59	0	17	258
	*Cloeon*	741	3368	6202	5878	10,916	11,223	7856	3894	1752	9050
Heteroptera	*Cymatia*	0	6	296	112	150	14	197	159	49	0
	Gerridae	1107	57	180	4	176	1843	134	89	8	28
	*Ilyocoris* ^a^	40	374	1185	364	116	313	576	474	395	70
	*Microvelia*	146	0	40	0	0	6150	211	108	0	0
	*Notonecta*	289	22	0	12	416	237	80	89	27	285
	*Plea*	237	80	853	188	136	1119	148	564	2	102
Odonata	Aeshnidae^a^	12	112	40	64	74	904	46	24	20	62
	Coenagrionidae^a^	536	502	1107	742	202	4885	429	1360	256	96
	*Libellula*	20	44	145	210	36	0	0	101	78	75
	*Orthetrum*	910	11	0	0	0	40,995	5	0	0	0
	*Sympecma* ^a^	1070	32	137	0	0	455	180	100	0	0
	*Sympetrum* ^a^	1192	173	557	0	36	1933	187	197	0	30
Bivalvia	Sphaeriidae	0	0	0	0	192	19,500	0	0	0	678
Gastropoda	*Bithynia*	51	20	0	60	166	100	0	86	260	2404
	*Ferrissia*	0	712	0	40	0	156	197	0	224	0
	*Gyraulus*	6335	18,076	23,603	286	1264	30,937	5384	594	31	6120
	*Hippeutis*	0	0	0	114	0	0	0	0	502	0
	*Lymnaea*	126	58	0	0	364	0	285	0	0	356
	*Physella*	79,387	2172	3574	424	190	44,958	333	19	107	3
	*Potamopyrgus*	0	0	617	12	0	0	0	462	0	0
	*Radix*	7	0	70	28	0	388	10	1821	0	0
	*Stagnicola*	387	0	0	4	0	370	0	0	163	48
Oligochaeta	Oligochaeta	80	86	60	4	28	363	176	9	9	51
Relative total abundance of the above taxa		0.99	0.98	0.97	0.93	0.95	0.99	0.99	0.96	0.87	0.95

^a^ Prey identified in the faecal samples.

**FIGURE 3:**
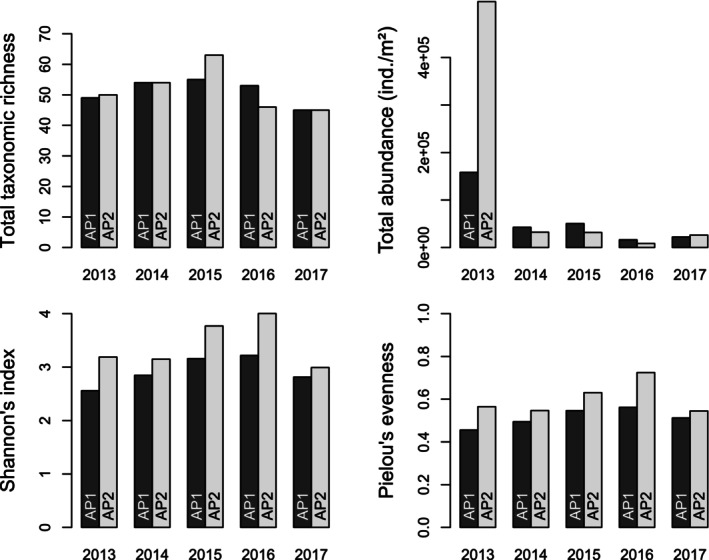
Temporal changes in macroinvertebrate community in the two acclimatisation ponds (namely, AP1 and AP2) between 2013 and 2017 on Woerr site, France: total taxonomic richness *S*, total abundance of macroinvertebrates (ind./m²), Shannon's index *H*′ and Pielou's evenness *E*.

From 2013 to 2017, the structure of the MI communities in both ponds was globally similar after successive turtle releases. Temporal changes in the values of the four metrics of community structure (total taxonomic richness, total abundance, Shannon's index and Pielou's evenness) remained quite similar between both ponds, albeit some differences could be observed: for instance, in 2013, the total abundance was particularly high for AP2 (Figure 3). Although total taxonomic richness remained very similar over time for both APs, both Shannon's index and Pielou's evenness were consistently higher for AP2 compared to AP1, regardless of the year (Figure 3). This difference was only significant for Pielou's evenness (Kruskal–Wallis test, *p* = 0.047). There was no significant effect of the years on the observed values of all four metrics (Kruskal–Wallis tests, *p* > 0.05 for all).

**FIGURE 4:**
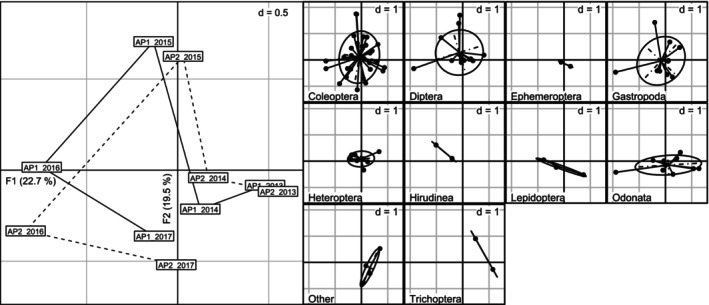
Correspondence analysis (CA) of the structure of the macroinvertebrate communities (log(*x* + 1)‐transformed densities, excluding rare taxa only observed once) found in the two acclimatisation ponds AP1 and AP2 on Woerr site, France, from 2013 to 2017. Left plot: Location of the ponds on the first factorial plane (F1 × F2). Labels indicate the pond and sampling year. Temporal changes are highlighted with lines, with a solid line for AP1 and a dashed one for AP2. Right plots: Location of the taxa on the first factorial plane, grouped per taxonomic group (*d* indicates grid size). One dot indicates one taxon. The taxonomic group ‘Other’ corresponds to taxa belonging to groups with only one taxon: Bivalvia, Megaloptera, Crustacea, and Oligochaeta.

Across all years, both acclimation ponds AP1 and AP2 exhibited similar MI community structures regardless of the respective number of turtles, as shown by the CA that explained > 40% of inertia on both first two axes (22.7% and 19.5% of the inertia, respectively; Figure 4). The main taxonomic groups, i.e., represented by the most abundant species (namely Coleoptera, Diptera, Gastropoda, Heteroptera and Odonata), were all globally located at the centre of the first factorial plane (F1 × F2; Figure 4). The trajectories of both APs on the first factorial plane between 2013 and 2017 were similar and consisted of a somewhat anticlockwise pattern around the centre of the plane, with no clear temporal evolution of the main structure of the observed communities. Variance partitioning confirmed the similarity between both ponds, with the sampling year explaining the majority of the variance (77.6%), whereas the pond identity only explained 5.1% of the variance (the unexplained residual variance accounted for 17.3%). The PERMANOVA confirmed the importance of the year (pseudo‐*F* = 4.484, *p* = 0.001) and the absence of an effect of the pond identity (pseudo‐*F* = 1.1792, *p* = 0.357) on the structure of the MI communities.

### 3.3 Turtle Diet and Biological Traits of Predated Versus Non‐Predated MIs

Among the 15 turtles faeces that were analysed, six samples were positive for MI, 10 samples were positive for amphibians (*Pelophylax*; water frogs) and 14 samples were positive for plants, while one sample (E095) was found negative for all taxa sequenced (Table 3). Faecal samples from five turtles (E031, E035, E043, E057, E091) contained DNA of both MI, amphibians, and plants; of one turtle (E083) DNA of both MI and plants (but no amphibians) and of three turtles (E002, E044, E080) plant DNA only. Regarding MIs, the obtained sequences belonged entirely to insects (no molluscs could be reliably neither identified nor sequenced in any samples), with the number of reads per taxa ranging from 34 to 523,647. On average, each turtle faecal sample contained 3.0 ± 1.3 different aquatic insect taxa, the most represented order being Odonata (taxonomic richness S = 6 taxa), followed by Coleoptera (S = 2, plus one terrestrial taxon) and Hemiptera (S = 2).

**TABLE 3:** Number of reads per assigned sequence in the faecal samples.

			Pond turtle individual	E002	E031	E035	E038	E040	E043	E044	E057	E069	E080	E083	E087	E091	E093	E095
			Carapace length (mm)	144.74	99.49	116.65	119.92	96.94	109.60	99.66	73.53	87.26	73.15	67.51	83.53	83.81	73.53	77.14
**Order**	**Family**	**Genera**	**Taxon**															
Coleoptera	Dermestidae	*Anthrenus*	*Anthrenus* sp. KY KLB‐2011	0	0	0	0	0	237	0	0	0	0	0	0	0	0	0
	Dytiscidae	*Hyphydrus*	*Hyphydrus ovatus*	0	0	0	0	0	0	0	0	0	0	0	0	58	0	0
	Hydrophilidae	*Enochrus*	*Enochrus testaceus*	0	0	0	0	0	0	0	0	0	0	0	0	70	0	0
Hemiptera	Naucoridae	*Ilyocoris*	*Ilyocoris cimicoides*	0	0	0	0	0	0	0	202,346	0	0	53	0	54	0	0
	Nepidae	*Ranatra*	*Ranatra linearis*	0	1,628,561	4345	0	0	0	0	0	0	0	0	0	0	0	0
Odonata	Aeshnidae	*Anax*	*Anax*	0	0	523,647	0	0	35,762	0	0	0	0	429	0	0	0	0
		—	Unidentified Aeshnidae	0	0	1768	0	0	24	0	0	0	0	0	0	0	0	0
	Coenagrionidae	*Coenagrion*	*Coenagrion pulchellum*	0	0	0	0	0	0	0	0	0	0	0	0	90	0	0
		*Ischnura*	*Ischnura elegans*	0	0	118,154	0	0	0	0	0	0	0	0	0	0	0	0
	Lestidae	*Sympecma*	*Sympecma fusca*	0	0	0	0	0	0	0	0	0	0	256	0	0	0	0
	Libellulidae	*Sympetrum*	*Sympetrum striolatum*	0	227	0	0	0	0	0	501	0	0	0	0	34	0	0
			Presence of amphibian DNA (*Pelophylax*)	No	Yes	Yes	Yes	Yes	Yes	No	Yes	Yes	No	No	Yes	Yes	Yes	No
			Presence of plants DNA	Yes	Yes	Yes	Yes	Yes	Yes	Yes	Yes	Yes	Yes	Yes	Yes	Yes	Yes	No

**FIGURE 5:**
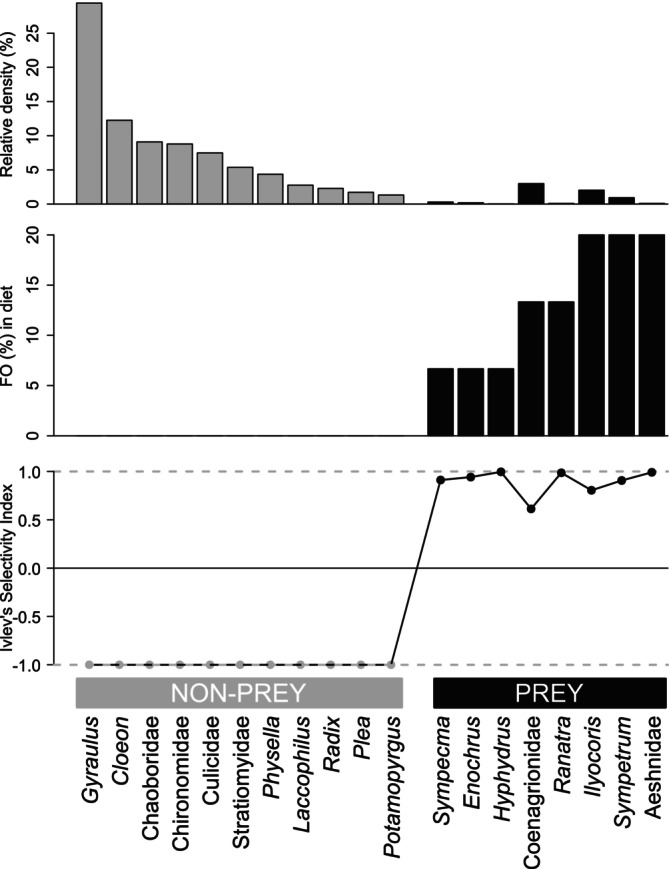
Top: Relative density of the most abundant macroinvertebrates (MI) taxa (i.e., with a mean relative density higher than 1%) and of the predated MI taxa in the acclimatisation ponds on Woerr site, France, in 2015. Middle: Frequency of occurrence (FO = number of turtle faeces containing a prey divided by the total number of faeces [*n* = 15]) of these taxa found in the faeces of 15 subadults European pond turtle released. Bottom: Associated Ivlev's electivity index for both non‐predated (in grey) and predated (in black) MI taxa.

Out of the most abundant MI taxa identified in the acclimatisation ponds in 2015 (i.e., with a mean relative density > 1% over the two ponds), only two out of the eight taxa identified in the turtles' faeces (hereafter referred as prey) exhibited high densities, with the two most abundant prey Coenagrionidae (pond damselflies) and *Ilyocoris* (naucorids) representing 3.0% and 2.0% of the overall density, respectively (Figure 5; top). These eight MI prey accounted for < 7% of the overall density. For these eight prey, the frequency of occurrence (FO) of each prey in the turtles' faeces ranged from 6.7% to 20.0%, the most frequently occurring prey in the faeces being *Ilyocoris* (creeping water bugs), *Sympetrum* (darters or meadowhawk) and Aeshnidae (hawkers, or darners). When considering Ivlev's electivity index of prey (ranging from 0.61 to 0.99), only one of these three prey had the maximum index (Aeshnidae, 0.99). Two other prey, namely *Hyphydrus* (diving beetles) and *Ranatra* (water scorpions or water stick‐insects), also exhibited the maximal electivity index value (0.99), despite having a lower FO (6.7% and 13.3%, respectively). Although Coenagrionidae were the most abundant prey in the ponds in 2015, they exhibited the lowest Ivlev's electivity index (0.61).

**FIGURE 6:**
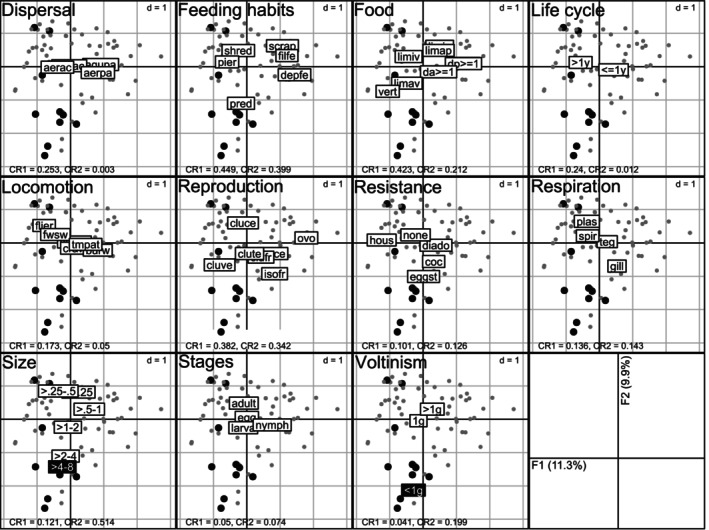
Ordination by fuzzy correspondence analysis of 11 biological traits of the macroinvertebrates (*n* = 76 taxa) identified in acclimatisation ponds (AP1 and/or AP2) on Woerr site, France, in 2015. The first 11 panels correspond to the distribution of taxa (identified by the dots) and trait categories (labels) of the biological traits on the F1 × F2 factorial plane. The black dots with a larger size correspond to the taxa sequenced in the 15 turtles' faecal samples whereas the grey dots correspond to taxa that were not found (not predated). Black labels indicate trait categories with coordinates along axis F2 lower than 1.426 (this threshold value was identified with a recursive partitioning analysis; see Figure S2). CR is the Correlation Ratio along axis F1 (CR1) or axis F2 (CR2): CR represents the percentage of variance explained by an axis to differentiate the categories of a trait and ranges from 0 (categories are not well separated along this axis) to 1 (this axis maximises the differentiation between all categories of a given trait) (Chevenet et al. 1994). Numbers on the last panel (bottom‐right) give the percentage of variance explained by each factorial axis. Full names of the trait modalities are given in Table S1. d indicates grid size.

The biological characteristics of the prey were identified based on a joint analysis of the biological traits of all MI (prey and non‐prey) taxa (Figure 6). The axes F1 and F2 of this FCA explained 11.4% and 9.9% of the total variance, respectively. Prey mainly clustered together at the bottom of the F1 × F2 factorial plane (Figure 6). A recursive partitioning analysis confirmed a significant difference between the coordinates of prey and non‐prey along axis F2 (*p* = 0.003; Figure S2). Two modalities of two different traits exhibited coordinates along axis F2 lower than the threshold detected via the recursive partitioning: prey were associated with a large maximum size (size between 4 and 8 cm; label: ‘> 4–8’) and a semi‐voltine reproduction (i.e., having one generation less often than once per year; label: ‘< 1 g’) (Figure 6).

**FIGURE 7:**
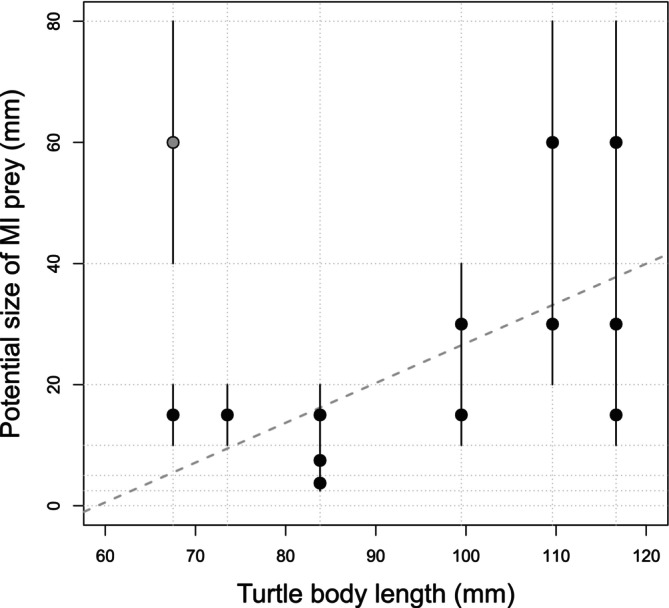
Relationship between body size of the six European pond turtles with macroinvertebrate (MI)‐positive DNA in their faeces and trait‐based potential size of corresponding MI prey (Table 3). Dots aligned vertically are associated with one single individual turtle that had DNA of MI prey of different potential sizes (the latter being the middle value of each class of prey potential size, i.e., ‘< 2.5 mm’ → 0.125 mm, ‘≥ 2.5–5 mm’ → 0.375 cm, ‘≥ 5–10 mm’ → 7.5 mm, ‘≥ 1–2 cm’ → 1.5 cm, ‘≥ 2–4 cm’ → 3 cm, and ‘≥ 4–8 cm’ → 6 cm). The vertical black bars are graphical representations of the range of each class of prey potential size. The grey dotted line corresponds to the regression line without the outlier (grey dot).

There was no significant relationship between the body size of turtles having preyed on MI and the maximum potential size of their prey (Spearman's rho = 0.348, *p* = 0.243). Yet, the relation became significant when removing the outlier corresponding to the smallest turtle (grey circle on Figure 7; Spearman's rho = 0.637, *p* = 0.025). Interestingly, excluding the above‐mentioned significant outlier turtle (E083; cf. Table 3), turtles with a body size < 90 mm consumed exclusively prey taxa having a potential size < 20 mm, whereas larger turtles could consume prey with a larger potential size.

**FIGURE 8:**
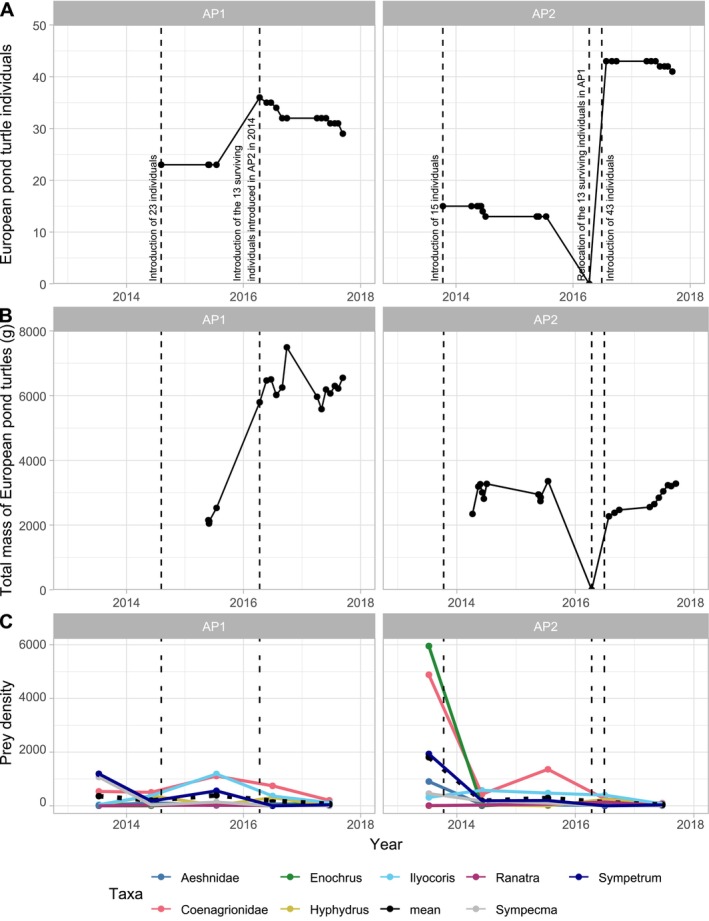
Number (top), total biomass (middle) of European pond turtles and mean density of macroinvertebrate prey (bottom) in the two acclimatisation ponds (AP1, AP2) on the Woerr site, France, between 2013 and 2017. Vertical lines indicate the translocation events of turtles (Table 1). Ticks for years are placed at the 1 January of a given year.

Regarding the MI taxa predated by turtles, average densities of all prey, when pooled together in 2013 (prior to the first turtle release), were higher in AP2 than in AP1 (1806 and 358 ind./m^2^, respectively; Figure 8). Then after, from 2014 to 2017, prey densities appeared to be similar in both ponds (Figure 8). Over the whole study period (2013–2017), Coenagrionidae (Odonata) and *Ilyocoris* (Naucoridae, Heteroptera) were the most abundant prey, with median densities equal to 519 (AP1) and 429 (AP2) ind./m^2^ for Coenagrionidae, and equal to 364 (AP1) and 394 (AP2) ind./m^2^ for *Ilyocoris*. *Sympetrum* (Odonata) remained also quite abundant over time (median equal to 173 and 187 ind./m^2^, respectively for AP1 and AP2). *Sympecma* (Odonata) were also somewhat abundant in AP2 (median density equal to 100 ind./m^2^), but not as much in AP1 (32 ind./m^2^). For all other prey, abundances remained comparatively lower, with a median density always lower than 100 ind./m^2^.

## 4 Discussion

In this study, we investigated the MI communities in two newly created, man‐made ponds concurrently with the diet of captive‐bred European pond turtles that were translocated there. We aimed at assessing whether turtles were operating as generalist feeders and if successive reintroductions of turtles would affect the overall MI communities by testing the prediction based on the optimal foraging theory that turtles would select larger prey for optimizing their food intake.

### 4.1 MI Communities Before and After Turtle Reintroduction Events

Both study ponds were man‐made and created in 2012, i.e., 1 year before the first turtles were released. In summer 2013, the MI communities found in both ponds were already quite species‐rich and abundant, confirming that MI colonisation of newly created ponds happens fast (e.g., Layton and Voshell 1991; Kuranchie et al. 2021). This was also confirmed by Ruhí et al. (2009), who showed that 50% of species in newly created ponds were already present 1–2 months after creation. Williams et al. (2007) reported that MI species richness reaches a plateau in three to 4 years. Both ponds exhibited communities largely dominated in abundance by ubiquists, active dispersers, and coloniser taxa, such as Diptera (e.g., Chironomidae, Chaoboridae) and Ephemeroptera (genera such as *Caenis* and *Cloeon*), consistent with previous studies of pond colonisation (Layton and Voshell 1991; Ruhí et al. 2009). In our study case, the close proximity of both ponds and the high similarity in their construction may explain their very similar MI communities since 2013. Faunistic groups frequently found in lentic habitats, such as Coleoptera, Heteroptera, and Odonata (Fairchild et al. 2000; Layton and Voshell 1991), were already abundant in 2013. Molluscs, such as Sphaeriidae (Bivalvia) and Gastropoda genera such as *Bithynia*, *Gyraulus* and *Physella*, were also quite abundant in 2013.

A first key finding of our study is that the MI communities found in both ponds changed through time, yet remained highly similar between ponds (both in terms of taxonomic richness, diversity, evenness and density; Figure 3) regardless of the year and successive translocations of varying numbers of turtles. Albeit mean densities of prey decreased in both basins between 2015 and 2017, turtle populations were not simultaneously reinforced during this period in both basins. In another anecdotal observation, the abundance of *Sympetrum* (darters or meadowhawks) decreased between 2013 and 2014 in both ponds, although by that time, turtles only occurred in one pond (AP2). Finally, the two prey that were the most abundant in the ponds (the pond damselflies Coenagrionidae and the creeping water bug *Ilyocoris*) exhibited synchronous temporal changes in abundance in AP1, with an increase from 2013 to 2015 and then a decrease until 2017. However, in AP2, these two prey exhibited highly different patterns in abundance changes from 2013 to 2015 before a synchronous decrease, seemingly unrelated to successive turtle reintroduction events. Overall, such an absence of major changes in the structure (composition and abundances) of the MI communities after the colonisation step was consistent with observations from other studies (e.g., Granath et al. 2024; Solimini et al. 2003), at least in the absence of extreme events. Finally, the absence of a significant effect of the total abundance or biomass of turtles in the ponds on MI taxonomic richness and diversity in both ponds (Figures 3 and 8) suggests that the turtles had no detectable effects on the MI community, at least during the 4 years following their release. The expansive submerged vegetation cover in the ponds may alleviate the predator effect on the MI community (e.g., Diehl 1992), for example by providing refuges. However, one should mention the only major difference in MI communities change (in terms of both global metrics, Figure 3, and prey density, Figure 8) between the two ponds occurred between 2013 and 2014, i.e., after the very first event where the largest turtles were released, as all metrics showed a dramatic decline in AP2 (where the first release occurred) compared to AP1 (Table 1, Figure 8).

We thus conclude that the reintroduction of European pond turtles did not affect the establishment and the dynamics of the MI communities, which seem to follow successional changes independent of the turtle population. However, the very first event where the largest turtles were released was associated with a sharp decline in MI community metrics. Considering that the duration of our study is of a similar time scale as the dynamics of MI settlement in newly created ponds (Ruhí et al. 2009; Williams et al. 2007), further monitoring is required for assessing potential long‐term effects of turtle reintroductions on the system, and the effects of the size of the released turtles on the MI communities.

### 4.2 European Pond Turtle Feeding Ecology and Selectivity

In our study, turtles that were sampled showed a diversified diet, including plants (14 out of 15 turtles), amphibians (*Pelophylax*, for 10 turtles) and MIs (only insects, mainly Odonata, Coleoptera and Hemiptera, for six turtles). This is consistent with previous studies that reported a diversified diet in the European pond turtle that feeds on plants and tadpoles when they are most numerous and/or easily attainable (Ayres et al. 2010; Çiçek and Ayaz 2011; Ducotterd et al. 2020; Ottonello et al. 2005, 2018). In our study, the relatively low number of turtles shown to have preyed on MIs may result from the fact that these turtles, as opportunistic feeders, preferably prey on non‐mobile food items, such as plants and amphibians, for which the biomass is generally high in summer when the faecal samples were taken. We thus advocate for further studies on the seasonal variability of the diet of European pond turtles (e.g., see Ducotterd et al. 2020). We may also advocate for combining DNA analyses with other complementary approaches, such as isotopic analyses, to better describe the diet of European pond turtles, especially the importance of each food item among trophic levels. Nevertheless, our results confirm that regardless of whether they are wild or captive‐bred individuals, the European pond turtle is a true generalist feeder.

A second key finding of our study revealed that the MI taxa consumed by European pond turtles (hereafter referred as prey) share similar key biological traits: they have a large potential size (relative to other MIs), a semi‐voltine life cycle (i.e., having a generation longer than 1 year), and are by far not the most abundant (< 7%) of the overall MI taxa occurring in the ponds (Figures 5 and 6). Both first two traits are highly correlated, as MIs with longer life cycles achieving larger body sizes (Usseglio‐Polatera et al. 2000), which may also contribute to their lower abundance relative to other (non‐prey) MI taxa. Three MI taxa, namely Aeshnidea (hawkers or darners), *Hyphydrus* (diving beetles) and *Ranatra* (water scorpions or water stick‐insects) occur in the turtles´ diet in higher proportions than they actually occur in the ponds, as indicated by their Ivlev's electivity index close to 1, suggesting that turtles actually select these large body‐sized prey. Such selection for large prey appears to be mediated by the turtles´ body size: our results suggest a threshold in turtle size (~90 mm body length) below which prey larger than 30 mm are not found in their diet (Figure 7). In other words, turtles larger than 90 mm appear to be able to feed on prey having a potential size up to 60 ± 20 mm (i.e., up to half their size). While DNA metabarcoding data cannot distinguish the prey stage (i.e., egg, larvae, adult), it is most likely that turtles mainly feed (i) on Aeshnidea larvae, that are aquatic predatory soft bodied organisms mainly fixed on the vegetation, whereas adults are aerial; (ii) on *Ranatra* individuals regardless of their instar stage, as they are slow‐moving organisms; and (iii) on *Hyphydrus* larvae, since adults are rapid swimming predators. Turtles may also feed occasionally, if not exceptionally, on remains or dead MIs, which could explain the detection of a large prey in the faeces of the smallest turtle (E083, Figure 7).

We thus conclude that the European pond turtle is a generalist feeder, yet turtles select prey of larger potential size, in accordance with our initial prediction based on the optimal foraging theory (MacArthur and Pianka 1966). Selection for larger prey occurs at the scale of the MI community and may be mediated by the body size of individual turtles, albeit our study failed to robustly demonstrate this aspect due to small sample size. These results advocate for further research investigating such potential size‐mediated and/or ontogenetically driven foraging strategy.

Finally, our study shows that the reintroduction of European pond turtles leads to an additional higher trophic level in the food web of the existing ecosystem, which, however, does not impair the trajectories of the MI communities during the 5 years following the first turtle releases. This may result from the low predation pressure exerted on the system by the small number of pond turtles that were released during this study period. This also advocates for long‐term monitoring of ecosystems centred on the European pond turtle, especially in the case of conservation initiatives such as reintroductions, for providing science‐grounded knowledge for the most appropriate management and decision making.

## Author Contributions

**Albin Meyer:** conceptualization (equal), data curation (equal), formal analysis (equal), methodology (equal), supervision (equal), validation (equal), visualization (equal), writing – original draft (equal), writing – review and editing (equal). **Corinne Grac:** conceptualization (equal), data curation (equal), formal analysis (equal), funding acquisition (equal), investigation (equal), methodology (equal), project administration (equal), resources (equal), supervision (equal), validation (equal), visualization (equal), writing – original draft (equal), writing – review and editing (equal). **Frédéric Labat:** conceptualization (equal), data curation (equal), formal analysis (equal), investigation (equal), methodology (equal), resources (equal), supervision (equal), validation (equal), visualization (equal), writing – original draft (equal), writing – review and editing (equal). **Johannes Meka:** formal analysis (equal), investigation (equal), methodology (equal), validation (equal), writing – original draft (equal), writing – review and editing (equal). **Karina A. E. van der Zon:** formal analysis (equal), investigation (equal), methodology (equal), validation (equal), writing – original draft (equal), writing – review and editing (equal). **Kathrin Theissinger:** conceptualization (equal), data curation (equal), formal analysis (equal), funding acquisition (equal), investigation (equal), methodology (equal), project administration (equal), resources (equal), supervision (equal), validation (equal), visualization (equal), writing – original draft (equal), writing – review and editing (equal). **Jean‐Yves Georges:** conceptualization (equal), data curation (equal), formal analysis (equal), funding acquisition (equal), investigation (equal), methodology (equal), project administration (equal), resources (equal), supervision (equal), validation (equal), visualization (equal), writing – original draft (equal), writing – review and editing (equal).

## Conflicts of Interest

The authors declare no conflicts of interest.

## Supporting information


Data S1.

## Data Availability

All data and the corresponding R script are available online: https://github.com/Albin‐Meyer/emys‐dna‐invertebrates‐diet.
