# Supplementary material for: Testing the Optimal Foraging Theory in a Generalist Feeder: The Case of Reintroduced European Pond Turtles and Its Impact on Macroinvertebrates Communities
Source: Ecol Evol. 2025 Aug 13;15(8):e71823. doi: 10.1002/ece3.71823 (PMC12349971; doi:10.1002/ece3.71823)
Supplement: Supplementary file 1 — Data S1. [file ECE3-15-e71823-s001.docx]

**Supplementary material**

Figure S1 Normalised principal component analysis (PCA) of the biometry of the pond turtles individuals which faecal samples were taken from. Left figure: correlation circle on the first factorial plane, F1xF2. Right figure: projection of the individuals on the factorial plane F1xF2.

Figure S2 Recursive partitioning between potential prey (P; dark grey) and the other taxa (NP, non-prey; light grey), based on the position of all taxa on the first 10 axes of the FCA presented in Figure 6. Only the coordinates on axis F2 could significantly discriminate between prey and non-prey.

Table S1 List of traits and their respective trait categories. Codes are used on Figure 6.

| Trait | Full name of the trait | Trait category | Code |
| --- | --- | --- | --- |
| Size | Maximal potential size | ≤ .25 cm | <=.25 |
|  |  | > .25-.5 cm | >.25-.5 |
|  |  | > .5-1 cm | >.5-1 |
|  |  | > 1-2 cm | >1-2 |
|  |  | > 2-4 cm | >2-4 |
|  |  | > 4-8 cm | >4-8 |
| Life cycle | Life span | ≤ 1 year | <=1y |
|  |  | > 1 year | >1y |
| Voltinism | Voltinism | < 1 gen/yr | <1g |
|  |  | 1 gen/yr | 1g |
|  |  | > 1 gen/yr | >1g |
| Stages | Aquatic stages | egg | egg |
|  |  | larva | larva |
|  |  | nymph | nymph |
|  |  | adult | adult |
| Reproduction | Reproduction strategy | ovoviviparity | ovo |
|  |  | isolated eggs, free | isofr |
|  |  | isolated eggs, cemented | isoce |
|  |  | clutches, cemented or fixed | cluce |
|  |  | clutches, free | clufr |
|  |  | clutches, in vegetation | cluve |
|  |  | clutches, terrestrial | clute |
| Dispersal | Dispersal technique | aquatic passive | aqupa |
|  |  | aquatic active | aquac |
|  |  | aerial passive | aerpa |
|  |  | aerial active | aerac |
| Resistance | Resistance forms | eggs, statoblasts | eggst |
|  |  | cocoons | coc |
|  |  | housings against desiccation | hous |
|  |  | diapause or dormancy | diado |
|  |  | none | none |
| Respiration | Respiration techniques | tegument | teg |
|  |  | gill | gill |
|  |  | plastron | plas |
|  |  | spiracle | spir |
| Locomotion | Locomotion techniques | flier | flier |
|  |  | surface swimmer | sursw |
|  |  | full water swimmer | fwsw |
|  |  | crawler | craw |
|  |  | burrower | burw |
|  |  | temporarily attached | tmpat |
| Food | Food types | detritus < 1mm | det<1 |
|  |  | dead plant >= 1mm | dp>=1 |
|  |  | living microphytes | limip |
|  |  | living macrophytes | limap |
|  |  | dead animal >= 1mm | da>=1 |
|  |  | living microinvertebrates | limiv |
|  |  | living macroinvertebrates | limav |
|  |  | vertebrates | vert |
| Feeding habits | Feeding habits | deposit feeder | depfe |
|  |  | shredder | shred |
|  |  | scraper | scrap |
|  |  | filter-feeder | filfe |
|  |  | piercer | pier |
|  |  | predator | pred |
